# Management and Treatment of Traumatic Brain Injuries

**DOI:** 10.7759/cureus.30617

**Published:** 2022-10-23

**Authors:** Shivangi Jha, Prajakta Ghewade

**Affiliations:** 1 Medicine, Jawaharlal Nehru Medical College, Datta Meghe Institute of Medical Sciences, Wardha, IND; 2 Anatomy, Jawaharlal Nehru Medical College, Datta Meghe Institute of Medical Sciences, Wardha, IND

**Keywords:** ferroptosis, tms, neuromodulation, blood brain barriers, tbi models

## Abstract

Traumatic brain injuries (TBI) are one of the main reasons for death in recent years worldwide or globally. They are the number one cause of death for both civilians and military members. It affects how the brain functions and is currently one of the crucial concerns of global public health issues. TBI is increasing worldwide because of the increasing dependency on motorized vehicles and machinery. One of the reasons for TBI is the expanding human population. It is the major cause of death and disability in the world. In young adults around the world, it is the main cause of mortality and morbidity. Its complicated etiology and pathogenesis include primarily primary and secondary injury types. Neuroinflammation is also focused on TBI to be cured. The neuroprotection of the injured brain has received tremendous attention during TBI treatment. In this review, we will first discuss the definition of traumatic brain injury, its causes, and the symptoms experienced by patients of various age groups. Finally, treatment methods and advances in treatment will be discussed. In this review, the aftereffects of traumatic brain damage are also covered. Ferroptosis and choline phospholipids are also emphasized as important components of the treatment of traumatic brain damage in this review.

## Introduction and background

Traumatic brain injury (TBI) is a condition where brain dysfunction is caused by an outside force, usually a violent blow to the head. It is an unexpected injury that harms the brain. A hit, bump, or jolt to the head may be the cause. The damage to the brain can be closed or open. It can happen when something pierces or penetrates the skull. It can also be referred to as a penetrating injury. Sudden head trauma can also cause traumatic brain damage [1]. TBI is a significant cause of death and disability for many young adults around the world. TBI can result in severe and challenging-to-treat long-term post-concussive symptoms such as depression and headaches.

Types and grades of TBI

There are several types and grades of TBI. Concussions are one of the most prevalent types of TBI. Every year, three out of four TBIs are concussions. People who suffer from a mild traumatic brain injury (mTBI) may suffer from confusion for about a day, which is different from attention and memory problems. Modest TBI is the second level of TBI. Less than 30 minutes of loss of consciousness are common with this kind of brain injury. Confusion may last for about a week. Another type of traumatic brain damage is severe TBI. With this type of injury, one can lose consciousness for about a full day. Uncomplicated TBI is another type in which a head CT or brain MRI is expected, regardless of whether it is mild, moderate, or severe. TBI in which the head CT/brain MRI shows changes such as hemorrhage is referred to as complicated TBI. Most TBIs are closed type. A closed TBI means that an outside force causes a blow or impact to the head that does not penetrate the skull. This blow injures the brain and causes it to swell. Some TBIs, however, are the open variety. The majority of medical professionals refer to an open TBI as a penetrating TBI. A bullet, knife, or other object penetrating the skull causes this damage. Brain tissue may be directly damaged when the object enters the brain [2]. 

Causes of TBI

There are several causes of TBI. The growing population and increasing dependence on motorized vehicles and machinery are one of the causes of increasing TBI cases. Stress is one of the reasons that lead to TBI. A person with significant anxiety may experience a traumatic brain injury or will eventually do so. A sudden blow to the head can cause traumatic brain injury. A bullet or any other object that penetrates the brain or skull can directly damage the brain and cause traumatic brain injury. A sudden fall from a height can also result in traumatic brain injury. It includes falls from a ladder, stairs, etc. Traumatic brain injury can result from violent behaviors including child abuse, domestic violence, and gunshot wounds. Traumatic brain damage can also result from sports injuries. This sort of injury includes a ball strike to the head. TBI is frequently brought on by explosive blasts in active-duty military personnel. Traumatic brain injuries can also result from penetrating wounds and hard impacts to the head from objects like splinters or debris. Traumatic brain injuries can also result from automobile collisions. TBI is frequently brought on by collisions with cars, motorcycles, or bicycles, as well as any injured pedestrians in such incidents [3].

## Review

Traumatic brain injuries have come into focus in recent years. It is a group of heterogeneous manifestations of a disease with high neurological morbidity [4]. The neurological components of TBI are primarily discussed in this review. The impacts of the blood-brain barrier (BBB) and traumatic brain damage are also mentioned, along with the effects of choline-containing phospholipids and ferroptosis on the treatment of TBI. Additionally, various age groups' experiences with traumatic brain damage are explored, as well as their symptoms. There are certain risk factors for a traumatic brain injury for example children, particularly those who are newborns to four years old, are most at risk of developing traumatic brain damage. Young adults between the ages of 15 and 24. Adults 60 years of age and older. All age ranges of men.

With the advances in biotechnology and medicine, the understanding of the effects of TBI has enabled the distinction between primary and secondary brain injury [4]. Moreover, it has been found that the level of choline or the level of its metabolites increases during the acute and chronic phases of TBI due to excitotoxicity, ischemia, and oxidative stress; this may help in diagnosing the prognosis and severity of TBI [5]. Several studies have shown that the apoptotic mechanism contributes to the pathology of TBI.

Symptoms

The physical and neurological repercussions of traumatic brain injury can be extensive. Some indications or symptoms could show immediately, while others might take a week or more to manifest. Mild traumatic brain injury symptoms and signs can include physical, sensory, cognitive, behavioral, or mental symptoms, etc. Brief information on each symptom is given below.

Physical Symptoms

Headache, nausea or vomiting, exhaustion or drowsiness, difficulty speaking, dizziness, or issues with balance are only a few of the physical symptoms. These are some of the common physical symptoms of TBI. Some of these effects may come on quickly, others may take time, and others may become a lasting problem [6].

Sensory Symptoms

TBI can lead to persistent sensorimotor and cognitive deficits, including long-term impaired sensory processing. Sensory problems include ringing in the ears, vision abnormalities, impaired vision, having a bad taste on your tongue or having trouble smelling. These symptoms usually occur as sensory symptoms in patients suffering from TBI [6].

Cognitive, Behavioral, or Mental Symptoms

Awareness loss lasts from a few seconds to a few minutes. There may not always be a loss of consciousness, but rather a feeling of lightheadedness, bewilderment, or disorientation. Issues with memory or concentration, including memory loss, lack of focus, etc. Additionally, people with traumatic brain damage may have mood changes and mood-swinging problems. Patients with traumatic brain injury can also experience emotions of depression, anxiety, or trouble sleeping as they have trouble sleeping soundly because of trauma or stress. Patients with TBI may experience long stretches of sleep than usual [6].

Children’s Symptoms

It's possible that young children and infants with traumatic brain injury can't explain their headaches, sensory problems, disorientation, or other symptoms. A kid with traumatic brain injury may exhibit symptoms including changes in feeding or nursing habits. Their eating habits are more unusual than normal when suffering from traumatic brain injury. Unusual or easy irritable habits are seen in a child that is they are often easily offended and irritable. They cry constantly and are unable to consolidate. Their ability to pay attention is also altered. They cannot pay attention properly if they are suffering from traumatic brain injury. As with adults, children with traumatic brain injury also have changes in their sleeping habits. They also experience seizures. They often do not feel well and are destined to have a sad and depressed mood. Drowsiness is often observed in them [7]. They lose interest in their favorite toys or activities they used to enjoy.

Neuroinflammation after traumatic brain injury

The pathogenesis of traumatic brain injury includes neuroinflammation. It may be one of TBI's most detrimental causes. After TBI, cases of both acute and chronic inflammation have been observed. Acute inflammation, however, can perpetuate prolonged, chronic, and toxic neuroinflammation as well as be neuroprotective by stimulating neurogenesis and nurturing spatial learning capacities as well as lowering the risk of infection [8]. Like macrophages, microglia can develop from monocytes into pro-inflammatory M1 microglia and/or anti-inflammatory M2 microglia, as well as from myeloid progenitor cells [9]. The majority of mononuclear phagocytes in the central nervous system (CNS) are microglia, which make up almost 10% of the CNS in adults [10].

Traumatic brain damage is linked to the BBB. Astrocytes, the cells that constitute the BBB, secrete chemokines that draw in undifferentiated monocytes. Simultaneously, the impairment of the neuronal cells makes the BBB permeable to peripheral nervous system (PNS) components [11]. DAMP-containing substances are released by the deteriorated neurons, which instigates the PNS to have another pro-inflammatory response [12].

The breakdown of membrane glycerophospholipids due to magnified phospholipase A2 (PLA2) activation incited by TBI results in the release of free fatty acids and lysophospholipids [13]. Because the derived fatty acids act as a substrate for the cyclooxygenases, which produce the eicosanoids, which further exaggerate the neuroinflammation, this PLA2 activity plays a more crucial role in the pathophysiology of TBI [14]. It is well known that lysophospholipids impair the membrane's fluidity and permeability [15]. Traumatic brain injury increases the brain's vulnerability to both enzymatic and non-enzymatic lipid peroxidation [16]. It is because the brain cannot regenerate, has a higher concentration of fatty acids, and needs more oxygen to carry out the proper metabolic functions [17]. 

Prevention

**Figure 1:**
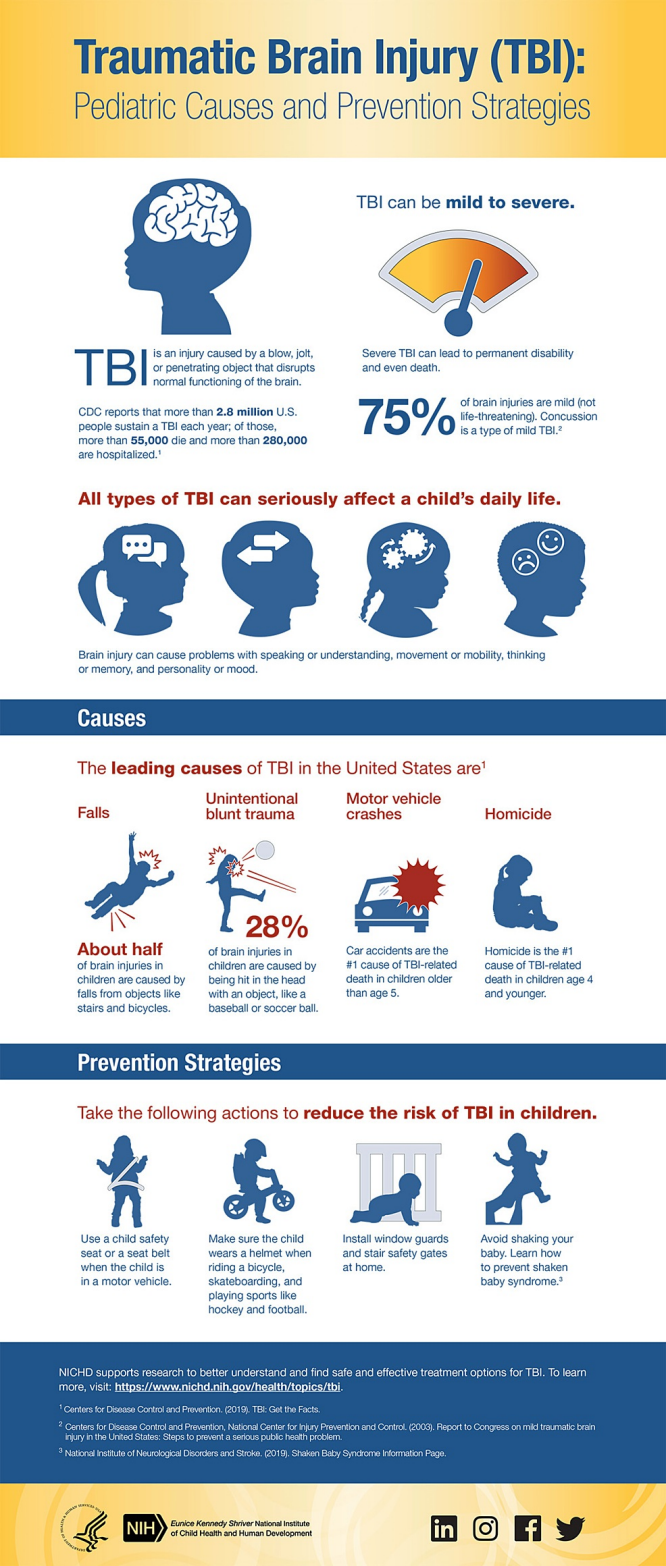
Prevention Strategies of Traumatic Brain Injury This figure has been taken from open access NIH website [19]

It is always said that prevention is better than cure, so now we know the prevention of traumatic brain injuries. We know the causes of TBI, and to some extent, we can prevent it (Figure 1). We can prevent TBI by reducing the dependency on motorized vehicles and machines. Severe stress and depression cause TBI, so there should be ways to reduce stress. Engaging in activities that keep one mind calm and cool so that stress would be reduced. Keeping safety during driving can help to reduce cases of TBI from road accidents. Such safety measures include like wearing a seat belt while in a vehicle, and wearing a helmet while on a bicycle, motorcycle, etc. One should never drive under the influence of alcohol or drugs. In sports, one should wear a proper helmet and headgear which are specific to the sport he or she plays. It should fit properly. Young children always are supervised i.e. they should be kept away at a safe distance from playgrounds with hard surfaces. On a construction site, one must always wear a hard hat as well as other protective equipment. In general, all the safety rules should be followed to prevent head injury [2,18].

Treatments

The management of traumatic brain injury is the next topic. TBI is a common neurological disorder that causes damage to the brain either permanently or temporarily. It is also a major cause of mortality in young and adults. So, treatment is required. The majority of TBI medical treatment focuses on symptoms. The major goals are maintenance and cessation of sedation and analgesic used for patients while treating intracranial hypertension and convulsive status epilepticus [20]. There is currently no treatment approach in use or available to stop the growth of secondary lesions in people [21]. Even though numerous pre-clinical investigations have been found to be effective pharmacological treatments to date, all phase III trials have been unsuccessful [22].

In contrast to other known forms of controlled cell death, ferroptosis is a distinct kind of regulated cell death (RCD) mode characterized by an excessive accumulation of reactive oxygen species (ROS) and iron-dependent lipid peroxide [23]. It is now recognized that ferroptosis plays a crucial role in the treatment of traumatic brain damage.

A crucial factor in determining the severity and prognosis of traumatic brain injury is choline and its metabolites [24]. According to Esele et al., after a craniocerebral transplant, the choline peak on an MRI correlates with myelin breakdown [25]. Additionally, during the chronic stages of TBI, Friedman et al. reported an increase in choline/creatine levels in the occipital grey matter, providing MRI evidence of cellular damage [26]. One of the most remarkable changes that take place following TBI is the elevated levels of free choline in the traumatized brain cortex and its surroundings [27]. The increased choline content in the chronic period might be the result of diffuse glial proliferation that is corroborated by elevated myoinositol levels that persists for months after damage [28]. Another explanation of this elevated choline in the chronic phase of TBI is the hyperosmolarity state of white matter, leading to the detection of increased choline [29].

It is well known that brain injuries lead to impairment of the BBB's integrity and function, which contributes to the loss of neuronal tissues and affects the response of the neuroprotective drugs. Therefore, one of the most promising strategies for preventing or minimizing the chances of neuronal inflammation, secondary brain injury, and acute neurodegeneration following TBI may involve stabilizing or protecting the BBB [30]. Additionally, it has recently appeared that patient-specific characteristics such as age affect the severity of TBI's effects in addition to the severity of mechanical factors [31]. Another crucial determinantal factor is the patient's sexual orientation [32].

Models used in the treatment of traumatic brain injury

Clinically relevant models are required for the development of novel TBI therapy alternatives. It is possible to imitate diverse injury processes, such as more localized or generalized damage, caused by focal brain contusion, or diffuse axonal injury (DAI), thanks to the range of models that have been used [33]. The controlled cortical impact (CCI) model, the lateral and central fluid percussion injury (FPI) models, and the weight drop/impact accelerations (I/A) model are probably the most widely utilized animal models in the treatment of TBI globally. Each of these models offers a unique set of advantages and disadvantages. Since TBI is not one disease, it is important to carefully analyze the clinical characteristics and complexity of human TBI when choosing the rodent TBI model following the current hypothesis, diagnosis, and impact of TBI [34]. The clinical TBI is replicated by FPI models without a skull fracture [35]. FPI can replicate all pathophysiological effects of human TBI, including intracranial hemorrhage, brain swelling, and progressive gray matter damage [36]. A thorough neuropathological analysis of the CCI TBI animals was conducted by Hall et al., who found that the damage could be extensive and include acute, cortical, hippocampus, and thalamic degeneration [37]. Penetrating ballistic-like brain injury (PBBI) is one of the models of TBI, which is caused by the transmission of projectiles with higher energy and a leading shockwave that produces a temporary cavity in the brain that is substantially larger than the bullet itself [38].

The astrocytes, the largest known cell type in the CNS, have a variety of physiological and pathological roles. It is now widely acknowledged that astrocytes contribute to both the development of TBI and tissue repair [39]. The activation of astrocytes has been shown to involve a wide variety of chemicals and signaling pathways, including essential life-supporting elements (such as ATP), regulatory hormones (such as gonadal steroids), injury-induced cytokines, and chemokines.

With promising findings for post-concussive depression and headache, transcranial magnetic stimulation (TMS) is one of the most researched neuromodulation techniques for mTBI (four for depression, four for headache, one for cognitive impairment, and two for global-post concussive symptoms) [40]. Additionally frequently utilized to treat individuals with intracranial brain injuries is hyperosmotic treatment. The use of omics technologies can also potentiate prognosis and diagnostics, as well as enhance our understanding of the injury mechanisms behind traumatic brain injury [41].

Methods of treatment

Injury Assessment

To treat an injury, it is important to assess it properly and know it well. So, it is the most crucial step in the treatment. The physician needs to know the severity of the injury to treat traumatic brain injury. They need to know the chances of full recovery or if the patient needs months of rehabilitation. In treating traumatic brain injury, three main treatments are used: medication, surgery, and exercises or therapies.

Medications

Diuretics can be used to treat TBI with medicine [42]. It is typically administered to lessen the amount of fluid in the tissues that get accumulated in the tissues as a result of traumatic brain injury. The medication also includes mannitol [43]. It is furosemide. Anticonvulsants are another type of drug used to treat TBI. They are typically used to prevent any further harm brought on by seizures. The medication that causes levetiracetam coma is carbamazepine. They are mainly employed in the situation of compressed blood vessels. Additionally, analgesics are also used as medication. In general, they are beneficial in reducing pain while treating traumatic brain injury. Many additional drugs, like propofol, pentobarbital, aspirin, etc., are also used to treat TBI [44].

Surgery

Surgery is one of the treatment methods for TBI in which the surgical removal of the affected parts is done, such as the skull, etc. It may be necessary in some cases of traumatic brain injuries. There are two types of injuries in such cases. Some surgeons prefer open brain surgery to remove the damaged part of the brain. In contrast, some surgeons choose closed surgery to remove the affected or damaged part of the brain without damaging any nerves. In such cases, surgeries can damage the brain, so it is essential to have good research on your treatment options before the surgery [3].

Physical Therapy

Therapies are also helpful in the treatment of TBI. They are equally important as the medication and surgical treatment methods of TBI. Treatment or simple therapies are usually meant for rehabilitation purposes. Alternative treatments are also available for those suffering from traumatic brain injuries. Chiropractors, osteopaths, and physical therapists can all provide therapy [45]. They can relieve the muscle spasms and contractions that are common after a traumatic brain injury. They can also improve circulation, which helps in decreasing inflammation. Osteopaths can also help to treat fractures and damage caused to the spine due to traumatic brain injury [46].

Exercises

It should be noted that exercises do not cure traumatic brain injury but only treat it. It is said that the activity does not increase blood circulation to the brain, which is a cause of brain inflammation. If exercises are performed correctly and regularly, there is an increase in the oxygen supply to the brain, which helps decrease or reduce the inflammation in the brain. Thus exercises are advised regularly though it does not directly increase blood circulation to the brain but increase oxygen supply to the brain which indirectly helps in reducing inflammation [47].

Acupuncture

It is used as an alternative treatment for traumatic brain injuries. Recent research has shown that it helps reduce pain in brain injuries. It is also observed that it helps supply blood to the brain tissues and reduces pain with the help of muscle contraction. It can also relieve the anxiety, depression, and insomnia that often affect patients after a traumatic brain injury. Thus this makes acupuncture a beneficial practice for the brain injury survivors.

After the effects of an injury

Traumatic brain injury is a serious illness or injury. It also leaves its mark on the person affected. Not everyone has the same problems or effects after a traumatic brain injury. It varies from person to person, or you can say it is unique for each person, and it depends on the severity of the traumatic brain injury. Some of the aftereffects have been mentioned. They can not concentrate or lower their concentration level. So they have trouble in concentrating or focusing. They have trouble in learning what they used to remember, which can be called memory difficulty [48]. They often face challenges potential with socializing. Their ability to speak is limited. They have problems with logical thinking and spatial awareness. Headaches are also a standard consequence of traumatic brain injury. In recovered patients of traumatic brain injury, a single head injury could lead to dementia in later life. The risk further increases with the increasing number of head injuries sustained by an individual. So, after recovery from traumatic brain injury, proper care is required.

## Conclusions

A catastrophic injury that results in death and impairment for people of all ages worldwide is a traumatic brain injury. Craniocerebral trauma is a complex phenomenon in which cellular damage develops as a result of structural stress, such as a blow to the head. A concussion in TBI can cause temporary confusion and headaches, while a severe TBI can be fatal. It does not include a stroke, brain infection, or brain tumor. It is necessary to cure and treat the disease. In this review, we have mentioned some of the management and treatment procedures that can be used or are already being used for TBI. Different animal models are also being used for the treatment of traumatic brain injury. There are still some research works going on on the treatment of TBI. From this review, we conclude that one should strictly follow traffic rules, sports safety rules, etc. to prevent brain injuries. Though there are now several types of research going on on the prevention of traumatic brain injury, it is always better to prevent a disease than to cure it.

## References

[1] Ownbey MR, Pekari TB (2021). Acute mild traumatic brain injury assessment and management in the austere setting-a review. Mil Med.

[2] Najem D, Rennie K, Ribecco-Lutkiewicz M (2018). Traumatic brain injury: classification, models, and markers. Biochem Cell Biol Biochim Biol Cell.

[3] Nitin Agarwal, MD MD, Rut Thakkar, Khoi Than, MD MD, FAANS FAANS (2022). Traumatic brain injury - causes, symptoms and treatments. AANS.

[4] Robinson CP (2021). Moderate and severe traumatic brain injury. Contin Minneap Minn.

[5] Bagri K, Kumar P, Deshmukh R (2021). Neurobiology of traumatic brain injury. Brain Inj.

[6] Karr JE, Iverson GL, Huang S-J, Silverberg ND, Yang C-C (2020). Perceived change in physical, cognitive, and emotional symptoms after mild traumatic brain injury in patients with pre-injury anxiety or depression. J Neurotrauma.

[7] Fordington S, Manford M (2020). A review of seizures and epilepsy following traumatic brain injury. J Neurol.

[8] Ziv Y, Ron N, Butovsky O (2006). Immune cells contribute to the maintenance of neurogenesis and spatial learning abilities in adulthood. Nat Neurosci.

[9] Shaked I, Tchoresh D, Gersner R (2005). Protective autoimmunity: interferon-gamma enables microglia to remove glutamate without evoking inflammatory mediators. J Neurochem.

[10] Colonna M, Butovsky O (2017). Microglia function in the central nervous system during health and neurodegeneration. Annu Rev Immunol.

[11] Das M, Mohapatra S, Mohapatra SS (2012). New perspectives on central and peripheral immune responses to acute traumatic brain injury. J Neuroinflammation.

[12] Toubai T, Mathewson ND, Magenau J, Reddy P (2016). Danger signals and graft-versus-host disease: current understanding and future perspectives. Front Immunol.

[13] Liu NK, Titsworth W, Xu XM (2009). Phospholipase A2 in CNS disorders: implication on traumatic spinal cord and brain injuries. Handbook of Neurochemistry and Molecular Neurobiology.

[14] Yui K, Imataka G, Nakamura H, Ohara N, Naito Y (2015). Eicosanoids derived from arachidonic acid and their family prostaglandins and cyclooxygenase in psychiatric disorders. Curr Neuropharmacol.

[15] Farooqui AA, Ong WY, Horrocks LA (2006). Inhibitors of brain phospholipase A2 activity: their neuropharmacological effects and therapeutic importance for the treatment of neurologic disorders. Pharmacol Rev.

[16] Bayir H, Kochanek PM, Kagan VE (2006). Oxidative stress in immature brain after traumatic brain injury. Dev Neurosci.

[17] Anthonymuthu TS, Kenny EM, Bayır H (2016). Therapies targeting lipid peroxidation in traumatic brain injury. Brain Res.

[18] (2022). Traumatic Brain Injury. https://medlineplus.gov/traumaticbraininjury.html.

[19] (2022). Infographic: Traumatic Brain Injury (TBI): Pediatric Causes and Prevention Strategies. Eunice Kennedy Shriver National Institute of Child Health.

[20] Geeraerts T, Velly L, Abdennour L (2018). Management of severe traumatic brain injury (first 24 hours). Anesth Crit Care Pain Med.

[21] Delage C, Taib T, Mamma C, Lerouet D, Besson VC (2021). Traumatic brain injury: an age-dependent view of post-traumatic neuroinflammation and its treatment. Pharmaceutics.

[22] Hiskens MI (2022). Targets of neuroprotection and review of pharmacological interventions in traumatic brain injury. J Pharmacol Exp Ther.

[23] Zhao Y, Huang Z, Peng H (2021). Molecular mechanisms of ferroptosis and its roles in hematologic malignancies. Front Oncol.

[24] Javaid S, Farooq T, Rehman Z (2021). Dynamics of choline-containing phospholipids in traumatic brain injury and associated comorbidities. Int J Mol Sci.

[25] Eisele A, Hill-Strathy M, Michels L, Rauen K (2020). Magnetic resonance spectroscopy following mild traumatic brain injury: a systematic review and meta-analysis on the potential to detect posttraumatic neurodegeneration. Neurodegener Dis.

[26] Friedman SD, Brooks WM, Jung RE, Hart BL, Yeo RA (1998). Proton MR spectroscopic findings correspond to neuropsychological function in traumatic brain injury. AJNR Am J Neuroradiol.

[27] Scremin OU, Jenden DJ (1991). Time-dependent changes in cerebral choline and acetylcholine induced by transient global ischemia in rats. Stroke.

[28] Ashwal S, Holshouser B, Tong K, Serna T, Osterdock R, Gross M, Kido D (2004). Proton spectroscopy detected myoinositol in children with traumatic brain injury. Pediatr Res.

[29] Lin AP, Liao HJ, Merugumala SK, Prabhu SP, Meehan WP 3rd, Ross BD (2012). Metabolic imaging of mild traumatic brain injury. Brain Imaging Behav.

[30] Sivandzade F, Alqahtani F, Cucullo L (2020). Traumatic brain injury and blood-brain barrier (BBB): underlying pathophysiological mechanisms and the influence of cigarette smoking as a premorbid condition. Int J Mol Sci.

[31] Newell E, Shellington DK, Simon DW (2015). Cerebrospinal fluid markers of macrophage and lymphocyte activation after traumatic brain injury in children. Pediatr Crit Care Med.

[32] Mollayeva T, Mollayeva S, Colantonio A (2018). Traumatic brain injury: sex, gender and intersecting vulnerabilities. Nat Rev Neurol.

[33] Petersen A, Soderstrom M, Saha B, Sharma P (2021). Animal models of traumatic brain injury: a review of pathophysiology to biomarkers and treatments. Exp Brain Res.

[34] Marklund N (2016). Rodent models of traumatic brain injury: methods and challenges. Methods Mol Biol.

[35] Thompson HJ, Lifshitz J, Marklund N, Grady MS, Graham DI, Hovda DA, McIntosh TK (2005). Lateral fluid percussion brain injury: a 15-year review and evaluation. J Neurotrauma.

[36] Graham DI, McIntosh TK, Maxwell WL, Nicoll JA (2000). Recent advances in neurotrauma. J Neuropathol Exp Neurol.

[37] Hall ED, Sullivan PG, Gibson TR, Pavel KM, Thompson BM, Scheff SW (2005). Spatial and temporal characteristics of neurodegeneration after controlled cortical impact in mice: more than a focal brain injury. J Neurotrauma.

[38] Williams AJ, Hartings JA, Lu XC, Rolli ML, Dave JR, Tortella FC (2005). Characterization of a new rat model of penetrating ballistic brain injury. J Neurotrauma.

[39] Li J, Wang X, Qin S (2021). Molecular mechanisms and signaling pathways of reactive astrocytes responding to traumatic brain injury. Histol Histopathol.

[40] Mollica A, Greben R, Oriuwa C, Siddiqi SH, Burke MJ (2022). Neuromodulation treatments for mild traumatic brain injury and post-concussive symptoms. Curr Neurol Neurosci Rep.

[41] Abu Hamdeh S, Tenovuo O, Peul W, Marklund N (2021). "Omics" in traumatic brain injury: novel approaches to a complex disease. Acta Neurochir (Wien).

[42] Jacobson HR (1987). Diuretics: mechanisms of action and uses. Hosp Pract (Off Ed).

[43] Patil H, Gupta R (2019). A comparative study of bolus dose of hypertonic saline, mannitol, and mannitol plus glycerol combination in patients with severe traumatic brain injury. World Neurosurg.

[44] Capizzi A, Woo J, Verduzco-Gutierrez M (2020). Traumatic brain injury: an overview of epidemiology, pathophysiology, and medical management. Med Clin North Am.

[45] Kane AW, Diaz DS, Moore C (2019). Physical therapy management of adults with mild traumatic brain injury. Semin Speech Lang.

[46] Dang B, Chen W, He W, Chen G (2017). Rehabilitation treatment and progress of traumatic brain injury dysfunction. Neural Plast.

[47] Perry SA, Coetzer R, Saville CW (2020). The effectiveness of physical exercise as an intervention to reduce depressive symptoms following traumatic brain injury: a meta-analysis and systematic review. Neuropsychol Rehabil.

[48] Paterno R, Folweiler KA, Cohen AS (2017). Pathophysiology and treatment of memory dysfunction after traumatic brain injury. Curr Neurol Neurosci Rep.

